# Combined Biosynthetic Pathway Engineering and Storage Pool Expansion for High-Level Production of Ergosterol in Industrial *Saccharomyces cerevisiae*

**DOI:** 10.3389/fbioe.2021.681666

**Published:** 2021-06-29

**Authors:** Zhi-Jiao Sun, Jia-Zhang Lian, Li Zhu, Yi-Qi Jiang, Guo-Si Li, Hai-Long Xue, Mian-Bin Wu, Li-Rong Yang, Jian-Ping Lin

**Affiliations:** Key Laboratory of Biomass Chemical Engineering of Ministry of Education, College of Chemical and Biological Engineering, Zhejiang University, Hangzhou, China

**Keywords:** ergosterol, lipid biosynthesis, *Saccharomyces cerevisiae*, metabolic engineering, two-stage fed-batch fermentation

## Abstract

Ergosterol, a terpenoid compound produced by fungi, is an economically important metabolite serving as the direct precursor of steroid drugs. Herein, ergsosterol biosynthetic pathway modification combined with storage capacity enhancement was proposed to synergistically improve the production of ergosterol in *Saccharomyces cerevisiae*. *S. cerevisiae* strain S1 accumulated the highest amount of ergosterol [7.8 mg/g dry cell weight (DCW)] among the wild-type yeast strains tested and was first selected as the host for subsequent metabolic engineering studies. Then, the push and pull of ergosterol biosynthesis were engineered to increase the metabolic flux, overexpression of the sterol acyltransferase gene *ARE2* increased ergosterol content to 10 mg/g DCW and additional overexpression of a global regulatory factor allele (*UPC2-1*) increased the ergosterol content to 16.7 mg/g DCW. Furthermore, considering the hydrophobicity sterol esters and accumulation in lipid droplets, the fatty acid biosynthetic pathway was enhanced to expand the storage pool for ergosterol. Overexpression of *ACC1* coding for the acetyl-CoA carboxylase increased ergosterol content from 16.7 to 20.7 mg/g DCW. To address growth inhibition resulted from premature accumulation of ergosterol, auto-inducible promoters were employed to dynamically control the expression of *ARE2*, *UPC2-1*, and *ACC1*. Consequently, better cell growth led to an increase of ergosterol content to 40.6 mg/g DCW, which is 4.2-fold higher than that of the starting strain. Finally, a two-stage feeding strategy was employed for high-density cell fermentation, with an ergosterol yield of 2986.7 mg/L and content of 29.5 mg/g DCW. This study provided an effective approach for the production of ergosterol and other related terpenoid molecules.

## Introduction

Ergosterol is the principal sterol in fungi cells and closely related to the membrane properties, such as the integrity, fluidity, permeability, and activity of membrane-bound proteins (Parks and Casey, 1995). It is also an important pharmaceutical precursor for the production of liposoluble vitamin D2 and sterol drugs (i.e., cortisone and progesterone) (Jasinghe and Perera, 2005; Karpova et al., 2016). In recent years, it has been found potential applications in the development of anticancer and anti-HIV drugs (Subbiah and Abplanalp, 2003; Kitchawalit et al., 2014). Due to the complex structure of ergosterol, its chemical synthesis is rather complicated and energy-consuming (Wu et al., 2012). Therefore, yeast fermentation has become the most attractive method for ergosterol production. *Saccharomyces cerevisiae* is a promising host due to its generally recognized as safe (GRAS) status, industrial robustness, and ease of genetic manipulation (Zhang et al., 2017). Moreover, *S. cerevisiae* with high flux through the mevalonate (MVA) pathway has been successfully engineered for efficient production of heterologous and native terpenoids, such as artemisinic acid (Westfall et al., 2012), α-santalene (Scalcinati et al., 2012), β-farnesene (Meadows et al., 2016), and squalene (Li et al., 2020). Various strategies have been employed to enhance ergosterol production in *S. cerevisiae*, including the screening of high ergosterol accumulation strains (He et al., 2000), genetic manipulation of the ergosterol biosynthetic pathway (Polakowski et al., 1999; He et al., 2003), and the optimization of fermentation conditions (Blaga et al., 2018).

**FIGURE 1:**
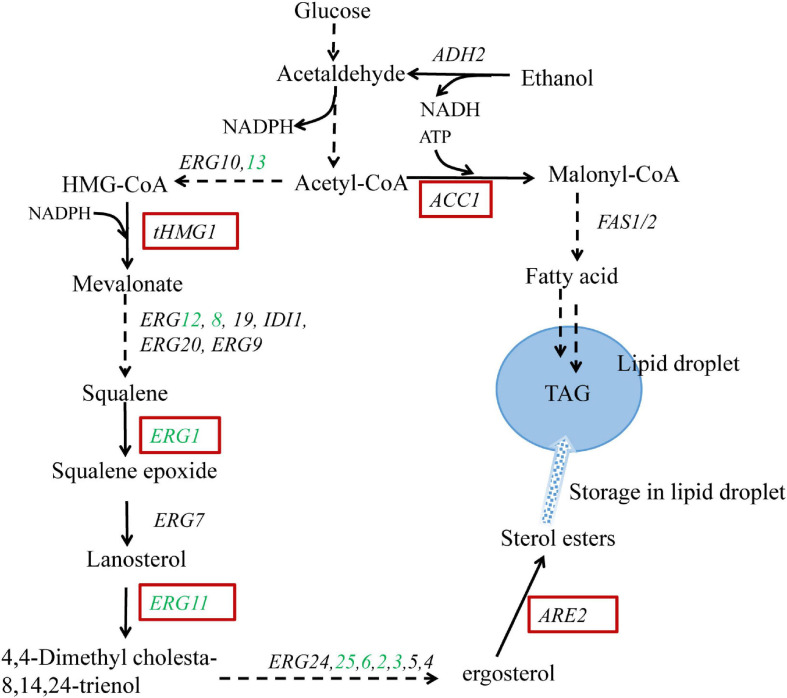
Schematic representation of the engineered metabolic pathways for ergosterol overproduction in *Saccharomyces cerevisiae*. Genes highlighted in red boxes are overexpressed; genes shown in green are upregulated via *UPC2-1* overexpression. The broken arrows represent multiple enzymatic steps, and the solid arrows represent a single enzymatic step. The metabolite triacylglycerol (TAG) is stored in the form of lipid droplets together with sterol esters. *tHMG1*: the catalytic domain of HMG-CoA reductase 1; *ARE2*: sterol acyltransferase; *ACC1*: acetyl-CoA carboxylase.

The ergosterol biosynthetic pathway in *S. cerevisiae* is rather complex with almost 30 enzymes involved and can be divided into two modules: squalene biosynthetic module (covering MVA pathway) and post-squalene biosynthetic module (Figure 1). Due to the cytotoxicity of the accumulation of excessive ergosterol, the ergosterol biosynthetic pathway and accordingly the intracellular ergosterol content is strictly regulated. Ergosterol biosynthesis is mainly controlled by feedback regulation at transcriptional, translational, and posttranslational levels (Espenshade and Hughes, 2007). In addition to the regulation of the biosynthetic pathway, the esterification of ergosterol is another regulatory mechanism (Jensen-Pergakes et al., 2001). Ergosterol and some of the steroid precursors can be stored as steryl esters (SE) in lipid droplets, serving as a sterol pool to maintain the balance of intracellular sterols.

Metabolic engineering efforts have been devoted to increasing the production of ergosterol in yeast. Polakowski et al. (1997) overexpressed the catalytic domain of HMG1 (3-hydroxy-3-methylglutaryl coenzyme A reductase 1) to significantly increase the supply of sterol precursor squalene (Polakowski et al., 1997). *ARE2* encoding a sterol acyltransferase was further overexpressed to enhance the esterification and accumulation of ergosterol in lipid droplet (Polakowski et al., 1999). In addition, overexpression of the positive regulators (i.e., ECM22, UPC2, or their mutants) has been proven to deregulate the sterol biosynthetic pathway, resulting in a significant increase in the production of terpenoids (Ro et al., 2006; Wang et al., 2018; Qiao et al., 2019). UPC2 (particularly the mutant UPC2-1) has been reported to upregulate sterol biosynthesis by specifically binding to the promoters of nine responsive *ERG* genes (*ERG1*, *ERG2*, *ERG3*, *ERG6*, *ERG8*, *ERG11*, *ERG12*, *ERG13*, and *ERG25*), most of which are involved in the post-squalene biosynthetic pathway (Yang et al., 2015). Moreover, Shin et al. (2012) reported that strains with upregulated fatty acid biosynthesis contained higher amounts of sterols, which was caused by increased transcription of the sterol biosynthesis related genes. Similarly, Ma et al. (2019) overexpressed key genes associated with fatty acid and triacylglycerol (TAG) biosynthesis to enlarge the cell-storage capacity for lipophilic products and accordingly promoted lycopene accumulation.

In this work, a combined strategy by coupling the increased metabolic fluxes toward ergosterol biosynthesis and the expanded storage capacity was employed to improve the production of ergosterol in the selected yeast strain. First, several *S. cerevisiae* strains was screened to determine a suitable host for ergosterol production. Then, the push and pull of ergosterol biosynthesis was engineered by increasing the supply of precursors and enabling the acylation of ergosterol. The storage capacity for hydrophobic products including ergosterol was enhanced by facilitating fatty acid biosynthesis. Moreover, to relieve the growth inhibition caused by ergosterol accumulation, ergosterol production was decoupled with cell growth by using a modified and auto-inducible *GAL* regulation system. Finally, high-cell density fermentation with a two-stage feeding strategy was performed in a 2 L bioreactor to fully explore the ergosterol production potential in this industrial *S. cerevisiae*.

## Materials and Methods

### Strains, Media, and Cultivation Conditions

Yeast strains used in this study are listed in Table 1. Yeast strains S1 (CICC 1746) and S2 (CICC 1306) were industrial strains for ergosterol production and obtained from China Center of Industrial Culture Collection. BY4741 and BY4742 were kind gifts from Professor Zhinan Xu (Zhejiang University, China). BY4741 genomic DNA was used for the amplification of *tHMG1, ARE2, UPC2*, and *ACC1*. *Escherichia coli Trans*-T1 (TransGen Biotech, China) was used as the host to construct, maintain, and amplify plasmids.

**TABLE 1:** Yeast strains used in this study.

**Name**	**Description**	**Source**
S1(CICC1746)	*MATa/*α	CICC
S2(CICC1306)	*MATa/*α	CICC
BY4741	*MATa his3Δ1 leu2Δ0 met15Δ0 ura3Δ0*	ATCC
BY4742	*MATα his3Δ1 leuΔ0 lys2Δ0 ura3Δ0*	ATCC
S1-tHMG1	S1, Δ*ho::P*_*PGK*1_-*tHMG1-T*_*ADH*1_	This study
S1-ARE2	S1, Δ*Ty4::P*_*PGK*1_-*ARE2-T*_*ADH*1_	This study
S1-tHA	S1-tHMG1, Δ*Ty4::P*_*PGK*1_-*ARE2-T*_*ADH*1_	This study
S1-AU	S1-ARE2, Δ*Ty3::P*_*ACT*1_-*UPC2-1-T*_*ADH*2_	This study
S1-tHAU	S1-tHA, Δ*Ty3::P*_*ACT*1_-*UPC2-1-T*_*ADH*2_	This study
S1-AUAC	S1-AU, Δ*P_ACC1_::P*_*TEF*1_	This study
S1-G	S1, Δ*gal80*	This study
S1-G-AUAC	S1-G, Δ*Ty4::P*_*GAL*1_-*ARE2-T*_*ADH*1_, Δ*ho::P*_*GAL*1_-*UPC2-1-T*_*ADH*2_, Δ*P*_*ACC*1_:: *P*_*GAL*1_	This study
		

Yeast and bacterial strains were stored in 25% glycerol at −80°C. *E. coli* was cultivated at 37°C in LB medium and ampicillin at 50 μg/mL was supplemented when necessary. Yeast strains were grown in YPD medium (1% yeast extract, 2% Peptone, and 2% glucose). When necessary, 200 mg/L G418 sulfate or 100 mg/L hygromycin B was added to the growth medium. For ergosterol production, the 2% glucose in YPD was replaced by 5% glucose.

### Plasmid Construction

All plasmids and primers used in this study are listed in Supplementary Tables 1, 2, respectively. Plasmid p42H-SpCas9 and pKan100-ADE2.1 (Lian et al., 2018), from Professor Huimin Zhao (University of Illinois at Urbana-Champaign, Urbana, IL, United States), were used for genome editing in yeast. Specific 20-bp targeting sequence of the gRNA molecule was introduced in the primers used for amplification of the entire pKan100-ADE2.1 plasmid by inverse PCR (Qi and Scholthof, 2008). The PCR product was then digested by *Dpn*I (TransGen Biotech, Beijing, China) and transformed into *E. coli.* The suitable target sequence was selected using E-CRISPR online tool (Heigwer et al., 2014). Donor DNAs for integration consisted of the expression cassettes and 50 bp homologous recombination arms. The expression cassettes of *tHMG1* and *ARE2* were prepared by fusing the promoters, open reading frames and terminators through overlap extension PCR (Heckman and Pease, 2007) using PrimeSTAR Max Premix (TaKaRa Bio, China). Donor DNAs containing the overexpression cassette of *UPC2-1* was amplified from p42H-*P_*ACT1*_-UPC2-1*. To construct the *TEF1* promoter donor DNAs used for the replacement of the native promoter of *ACC1*, the homologous arms were prolonged to 200 bp, and assembled with *TEF1* promoter by overlap extension PCR. As for the construction of the donor DNAs for *GAL80* deletion, the 460 bp upstream and 478 bp downstream fragments were amplified from the genome of strain S1 and pieced together using overlap extension PCR. The full-length donor DNA fragments were gel purified and cloned into the *pEASY*^®^-Blunt Simple Cloning Vectors (TransGen Biotech, Beijing, China). To construct the donor DNAs for integration with inducible expression of *ARE2*, *UPC2-1*, and *ACC1*, the constitutive promoters in the plasmid containing the relative donor DNAs was replaced by *GAL1* promoter via homologous recombination using ClonExpress II One Step Cloning Kit (Vazyme, Nanjing, China). The expression cassette of *UPC2* with *TEF1* promoter was first generated by replacing Cas9 in plasmid p42H-SpCas9, resulting in the construction of the plasmid p42H-*P_*TEF1*_-UPC2*. The pleiotropic mutation G888D of the regulation factor UPC2 was introduced by inverse PCR using plasmid p42H-*P_*TEF1*_-UPC2* as a template, generating p42H-*P_*TEF1*_-UPC2-1*. The plasmid p42H-*P_*ACT1*_-UPC2-1* was constructed by replacing the *TEF1* promoter of plasmid p42H-*P_*TEF1*_-UPC2-1* using ClonExpress II One Step Cloning Kit.

### Yeast Transformation and Strain Construction

CRISPR/Cas9 guided gene knock out, integration, and substitution were performed as previously described with some modifications (Lian et al., 2018). Yeast cells were transformed by the PEG/LiAc method (Gietz and Schiestl, 2007). The Cas9 expressing strains were constructed by transforming p42H-SpCas9 into the corresponding yeast strains. For the co-transformation of gRNA expression plasmids and donor DNAs into Cas9 expressing strains, heat shock time was prolonged to 90 min, and the yeast strains were recovered in 2 mL YPD/Hyg for 13 h to allow sufficient expression of the G418 resistance gene. Then the transformants were selected on YPD/Hyg+G418 plates and confirmed by colony PCR and DNA sequencing.

### Fed-Batch Fermentation

Single colonies were inoculated into 5 mL YPD medium and cultured at 30°C for 24 h and then transferred into 250 mL flasks containing 50 mL of YPD medium. After 20 h cultivation, two flasks of cultures were used to inoculate 0.9 L fermentation medium (10 g/L glucose, 10 g/L (NH4)_2_SO_4_, 8 g/L KH_2_PO_4_, 3 g/L MgSO_4_, 0.72 g/L ZnSO_4_.7H_2_O, 10 mL/L trace metal solution, and 12 mL/L vitamin solution) in a 2 L glass bioreactor (T & J-MiniBox, Shanghai, China). Fermentations were carried out at 30°C, and pH was controlled at 5 by automatic addition of 5 M NH_4_OH. Dissolved oxygen (DO) was maintained at >15% saturation by adjusting the agitation rate (300–1,000 rpm) and airflow rate (1–3 vvm). After the batch culture phase, a feeding solution containing 500 g/L glucose and 12 mL/L vitamin solution was used to achieve fast cell growth with the pseudo-exponential feeding model. For the last phase, ethanol was fed to the fermenter until the end of the fermentation to improve the intracellular accumulation of ergosterol. The feeding rate *F*_*S*_ during the pseudo-exponential phase was determined by the following equations (Zhao et al., 2016):

$$F_{s}=\left(\frac{\mu }{Y_{x/s}}+m\right)\cdot \frac{X_{0}V_{0}}{S}\cdot e^{\mu t}$$

where *X*_0_, *V*_0_, and *S* were the initial biomass density [g dry cell weight (DCW)/L], the initial culture volume (L), and the glucose concentration (g/L) in the feeding medium; *Y*_*X/S*_ was the yield of the cell on glucose (g DCW/g glucose); μ was the specific growth rate (h^–1^), m was the maintenance coefficient (g glucose ⋅ g DCW^−1^ ⋅ h^−1^), and *t* was the time (h) after starting the feeding. A predetermined specific growth rate of 0.13 h^–1^ was used to avoid overflow metabolism. The values of *Y*_*X/S*_ and m were 0.5 and 0.05, respectively, according to the previous study (Mendoza-Vega et al., 1994). The feeding rate was not corrected for the amount of ammonium hydroxide added or the total volume of the culture samples withdrawn from the fermenter. The feeding rate was adjusted every hour, according to the theoretical model.

### Quantitative Analysis

Cells growth was monitored by measuring optical density at 600 nm (OD_600_) using a visible spectrophotometer (721G, INESA, Shanghai, China). DCW was determined from plots of OD_600_ and DCW. The total ergosterol and squalene in yeast cells were extracted as previously described (Rodriguez et al., 2014) with some modification. As for ergosterol extraction, 500 μL of yeast cell culture was harvested and washed twice with sterile distilled water, 500 μL of alcoholic KOH solution [25% (w/v) in 50% EtOH] was added to the yeast pellets and mixed by vortexing for 1 min. Cell suspensions were then boiled for 1 h. After cooling on ice, ergosterol was extracted with 1 mL petroleum ether, followed by vigorous vortexing for 3 min. Four hundred microliter of petroleum ether (top) layer was collected and dried with a vacuum dryer. Dried samples were dissolved in 500 μL ethanol and analyzed by high-performance liquid chromatographic (HPLC), using a C-18 column (Hypersil BDS 5μm, 4.6mm × 250mm), with a UV detector at 280 nm. Methanol/acetonitrile (80: 20, v/v) was used as the mobile phase with an elution rate of 1 mL/min. As for the quantification of squalene, cells from 1.8 mL culture were collected and saponified in 600 μL alcoholic KOH solution followed by extraction with 600 μL petroleum ether. A total of 400 μL of the top layer was dried by vacuum dryer, then dissolved in 500 μL ethanol, and subjected for HPLC analysis using 100% acetonitrile as the mobile phase with an elution rate of 1.5 mL/min and a UV detector at 215 nm. All results were reported as the average of biological triplicates. Glucose and ethanol concentrations were determined off-line using a biosensor (SBA-40C; Biology Institution of Shandong Academy of Science, Jinan, China).

### RNA Isolation and Transcript Quantification

Real-time reverse-transcription PCR (qRT-PCR) analysis was performed to confirm the overexpression of the genes integrated into the genome. The RNAiso Plus Kit (TaKaRa, China) was used for the extraction of total RNA from the harvested yeast cells. The residual genomic DNA in RNA samples was digested by DNaseI (TransGen Biotech, Beijing, China). The cDNA templates were synthesized from the DNaseI treated total RNA using LunaScript^TM^ RT SuperMix Kit (NEB, China). Then quantitative PCR (qPCR) reactions were performed in a StepOne Plus Real-time PCR System (Applied Biosystems, United States) using Luna Universal qPCR Master Mix (NEB, China). The *ACT1* gene was selected as the internal control gene to normalize the amount of the total RNA in different samples. The relative transcriptional level for each gene was determined using the 2^–ΔΔCt^ method (Livak and Schmittgen, 2001).

## Results and Discussion

### Selection of Appropriate Yeast Strains for Ergosterol Production

**FIGURE 2:**
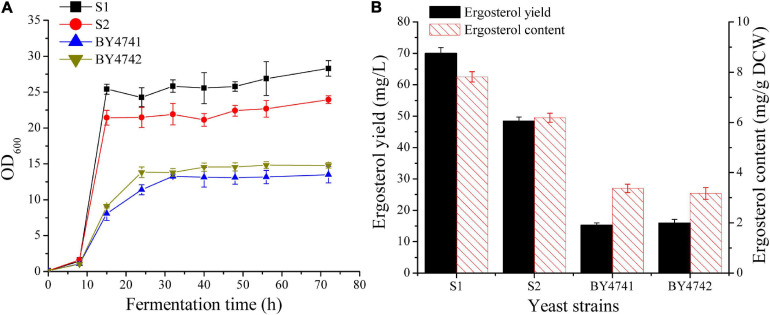
Comparison of cell growth and ergosterol production in wild-type *S. cerevisiae* strains. **(A)** Growth curves of different strains in 250 mL shake flasks with 50 mL of YPD medium containing 50 g/L glucose. **(B)** Ergosterol production in shake flasks by different strains after 48 h cultivation.

In *S. cerevisiae*, ergosterol biosynthesis is tightly controlled (Henneberry and Sturley, 2005). It is necessary to select a strain with a relatively high basal level of ergosterol for subsequent metabolic engineering studies. The two industrial diploid yeast strains [CICC1746 (S1) and CICC1306 (S2)] and two laboratory strains (BY4741 and BY4742) were first cultivated in 50 mL flask to investigate their growth characteristics (Figure 2A) and ergosterol contents (Figure 2B). For the two industrial strains S1 and S2, OD_600_ was increased rapidly during the first 15 h and finally reached up to 28 and 24. In contrast, the two laboratory strains grew gradually and their final OD_600_ was approximately 14. As shown in Figure 2, strain S1 achieved the best values of biomass and ergosterol productivities when compared with other strains. The ergosterol content for S1 (7.8 ± 0.2 mg/g DCW) was significantly higher than that for BY4741 (3.4 ± 0.2 mg/g DCW) and was slightly higher than that for S2 (6.2 ± 0.2 mg/g DCW). Taking biomass and ergosterol accumulation into consideration, strain S1 (69.9 ± 1.9 mg/L), was selected as the parent strain for further manipulation.

### Engineering of the Pull and Push of Ergosterol Biosynthesis for Enhanced Ergosterol Accumulation

Previous reports have determined that HMG-CoA reductase is a rate-limiting enzyme in the MVA pathway and is highly regulated (Rodwell et al., 1976). Overexpression of *tHMG1* can eliminate the feedback regulation, thus increasing carbon flux through the MVA pathway (Donald et al., 1997; Polakowski et al., 1997). This strategy has been successfully used before to boost the production of various isoprenoids in *S. cerevisiae* (Tokuhiro et al., 2009; Paddon et al., 2013; Xie et al., 2014; Qiao et al., 2019). As a first step toward an ergosterol-overproducing yeast strain, the *tHMG1* expression cassette (controlled by the *PGK1* promoter) was integrated into the chromosome of S1 at the *HO* site, resulting in the strain S1-tHMG1. As expected, its squalene production was obviously increased and reached to 10.8 ± 0.3 mg/L within 48 h flask fermentation, while the squalene content in reference strain S1 was too low to be detected (Supplementary Figure 1). Consistent with prior reports that overexpression of *tHMG1* leads to a reduction in growth rate (Donald et al., 1997), inferior growth was observed in the squalene overproducing strain S1-tHMG1 (Supplementary Figure 2A). The maximum cell density of the *tHMG1* overexpression strain was 18% lower than that of the wild type strain. Subsequent qRT-PCR analysis revealed that the transcriptional level of *tHMG1* in recombinant strain S1-tHMG1 was about 10-fold higher than that in the control strain S1 (Supplementary Figure 3). However, the increased content of squalene did not lead to the noticeable formation of ergosterol. Ergosterol production of strain S1-tHMG1 (8.2 ± 0.7 mg/g DCW) was almost the same as that of the parent strain S1 (Supplementary Figure 2B). Our result is consistent with previousl studies that ergosterol was not accumulated in *tHMG1* overexpression strains (Polakowski et al., 1997). This is because the post-squalene biosynthetic pathway was under tight transcriptional regulation and limiting the accumulation of ergosterol in yeast.

**FIGURE 3:**
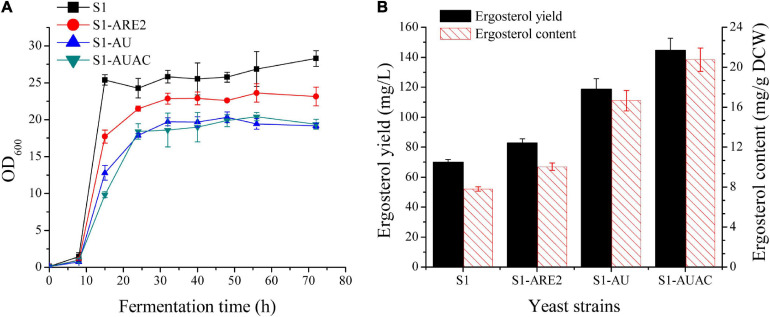
Effect of overexpression of *ARE2*, *UPC2-1*, and *ACC1* on cell growth and ergosterol production. **(A)** Cell growth curves and **(B)** ergosterol production of the strains S1, S1-ARE2, S1-AU, and S1-AUAC in 250 mL shake flasks with 50 mL of YPD medium containing 50 g/L glucose. Samples for ergosterol quantification was collected after 48 h cultivation.

In *S. cerevisiae*, ergosterol can be converted to SE and stored in lipid droplets (Yang et al., 1996; Polakowski et al., 1999). Esterification enhancement may be an efficient strategy in improving ergosterol accumulation in yeast. It has been found that the sterol acyltransferases ARE2 is mainly responsible for the esterification of sterols, especially ergosterol (Zweytick et al., 2000). Thus, *ARE2* expressing cassettes (controlled by the *PGK1* promoter) were inserted in the genomes of strain S1 and squalene accumulation strain S1-tHMG1, respectively, generating the strains S1-ARE2 and S1-tHA. Overexpression of *ARE2* increased ergosterol content to 10 ± 0.4 mg/g DCW, which was 28% higher than that of the parental strain (Figure 3B). As shown in Supplementary Figure 1, squalene content in strain S1-tHA was decreased by 62% compared with strain S1-tHMG1, suggesting that enhancing ergosterol esterification is beneficial to increase the metabolic flux from squalene to ergosterol. Ergosterol contents of S1-ARE2 and S1-tHA were determined to be nearly the same, indicating that enhanced supply of the precursor squalene didn’t promote the excessive synthesis of the final product ergosterol.

Since the enzymes involved in the post-squalene pathway are tightly regulated in yeast via transcriptional regulation, overexpression of positive regulators can be effective for metabolic engineering. UPC2 is a global transcription factor that positively regulates the transcription of genes involved in the ergosterol biosynthesis and facilitates exogenous sterol uptake under low oxygen conditions (Davies et al., 2005; Zavrel et al., 2013). The mutation of UPC2, UPC2-1(G888D), results in the constitutive activation of the transcription factor, leading to the upregulation of ergosterol synthesis pathway in yeast. Therefore, overexpression of *UPC2-1* is usually employed to improve the produciton of terpenoids. The well-known case is that the overexpression of *UPC2-1* in the engineered yeast resulted in significantly improved production of artemisinin precursors (Ro et al., 2006; Westfall et al., 2012). In this study, the *UPC2-1* expressing cassette under the control of a strong constitutive promoter (*P*_*ACT1*_) was inserted to the genome of S1-ARE2 and S1-tHA, resulting in the construction of strains S1-AU and S1-tHAU. The growth rate of strain S1-AU was further decreased when compared with S1-ARE (Figure 3B), indicating that overexpression of *UPC2-1* leads to metabolic burdens and growth inhibition. As shown in Figure 3B, 118 mg/L ergosterol yield with a content of 16.7 mg/g DCW was synthesized in strain S1-AU, which were 116.8 mg/L and 16.8 mg/g DCW in strain S1-tHAU (Supplementary Figure 2B). Overall, the overexpression of *tHMG1* failed to increase the production of ergosterol either individually or in combination with *ARE2* and/or *UPC2-1* overexpression. Therefore, *tHMG1* overexpression was not included for further optimization of ergosterol biosynthesis.

### Expanding Ergosterol Storage Pool via Enhancing Fatty Acids Biosynthesis

Sterols and lipids, both of which are stored in lipid droplets, play crucial roles in cell membrane fluidity and permeability (Figure 1). Under normal conditions, the relative ratio of these membrane components is very stable, and variation in the production of one component will affect the biosynthesis of the other (Aguilera et al., 2006). It has been revealed that the biosynthetic pathway of sterol is co-regulated to other pathways involved in the biosynthesis of lipids (Veen and Lang, 2005; Klug and Daum, 2014). Sun et al. (2016) showed that both β-carotene and ergosterol productions were significantly increased with the addition of oleic acid and palmitoleic acid to the cultures. Therefore, modulating fatty acid biosynthesis might benefit ergosterol production in the industrial yeast. Accordingly, the key enzyme ACC1 that determines the fatty acid biosynthesis was overexpressed in strain S1-AU by replacing the native promoter of *ACC1* with a strong constitutive promoter *P*_*TEF1*_, generating strain S1-AUAC. The growth curve of the S1-AUAC remained almost synchronous with that of S1-AU (Figure 3A). Chromosomal replacement of the *ACC1* promoter with *P*_*TEF*1_ led to an ergosterol content of 20.7 mg/g DCW, a 25% increase over that of S1-AU (Figure 3B). Ergosterol yield was increased from 118.5 to 144 mg/L. Analysis by qRT-PCR confirmed that the transcription levels of *ARE2*, *UPC2-1*, and *ACC1* in strain S1-AUAC were 5. 9-, 4. 6-, and 2.4-fold higher than those in the parent strain (Supplementary Figure 3).

Synchronization between sterol and lipid synthesis is essential for cells to maintain lipid homeostasis and adequate response to changes in environmental conditions (Guo et al., 2018; Scodelaro Bilbao et al., 2020). Shin et al. (2012) revealed that improvement of fatty acid biosynthesis induced a significant increase of the total sterols including zymosterol and ergosterol. More specifically, overexpression of *ACC1* significantly increased the expression level of *ERG11*, *ERG28*, *ERG5*, *ERG2*, and *ERG20*. Ma et al. (2019) revealed that increased fatty acid biosynthesis and TAG production could improve the cell-storage capacity for lipophilic compounds, which resulted in the overproduction of lycopene. The improved sterol storage caused by lipid droplet formation might also promote ergosterol accumulation. Our study showed that in addition to pathway engineering of ergosterol biosynthesis, expanding the storage pool by modulating the lipid metabolic pathway offers another promising approach to stimulate the production of ergosterol in yeast.

### GAL-Based Autonomous Induction System for Dynamic Regulation of Ergosterol Biosynthesis

Compared with the parent strain S1, although ergosterol production was significantly improved, severe growth inhibition was observed in recombinant strains overexpressing the selected genes under the strong constitutive promoters. This can be attributed to the increased metabolic burdens in the exponential phase and the cytotoxicity of free sterols on cells. In order to obtain high ergosterol-producing strain without growth defects, an alternative option is to separate biomass accumulation (cell growth) and ergosterol production into two-independent phases (Wu et al., 2016). This can be achieved by the introduction of inducible promoters that responds to bioprocess conditions (Xie et al., 2014). In this work, the galactose-inducible expression system, one of the most commonly used in yeast, was employed to control the expression of ergosterol overproduction related genes (Da Silva and Srikrishnan, 2012). Nevertheless, it is rather expensive to induce gene expression by galactose for industrial production. Fortunately, previous studies have shown that the disruption of *GAL80* resulted in transcriptional activation of genes under the control of *GAL* promoters without the presence of galactose when the glucose concentration is low. In other words, the target genes driven by *GAL* promoters can be repressed in the cell-growth phase when glucose concentration is high and automatically activated when glucose becomes low in the fermentation media (Xie et al., 2014).

**FIGURE 4:**
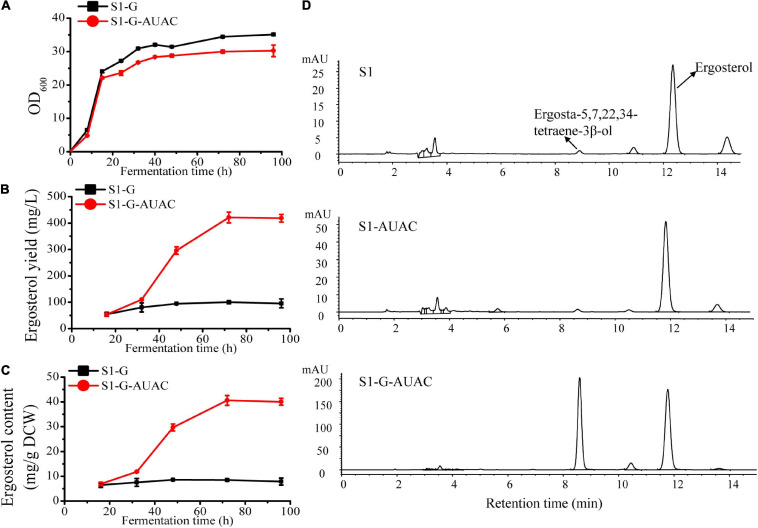
Effect of *gal80Δ-GAL1* promoter constructs on growth and ergosterol production. **(A)** Cell growth curves of strain S1-G-AUAC and the control strain S1-G; time course of **(B)** ergosterol yield and **(C)** ergosterol content of strains S1-G and S1-G-AUAC. Samples were collected at 16, 32, 48, 72, and 96 h during the flask fermentations. **(D)** Comparison of high-performance liquid chromatographic (HPLC) profiles of strains S1 (parent strain), S1-AUAC (constitutive overexpression of *ARE2*, *UPC2-1*, and *ACC1*), and S1-G-AUAC (inducible overexpression of *ARE2*, *UPC2-1*, and *ACC1*). All the strains were cultivated in 250 mL shake flasks with 50 mL of YPD medium containing 50 g/L glucose.

In the current study, S1-G was first constructed based on S1 by deleting *GAL80* to eliminate the dependency on galactose induction. With the *ARE2* and *UPC2-1* overexpression cassettes controlled by *GAL1* promoter integrated into the genome of S1-G and the native *ACC1* promoter replaced by *GAL1* promoter, S1-G-AUAC was constructed. As shown in Figure 4A, the growth of strain S1-G-AUAC was nearly the same as that of S1-G during the first 12 h of cultivation. At the later stage, slight growth inhibition was observed in S1-G-AUAC, probably due to the biosynthesis and accumulation of ergosterol. During the whole fermentation process, there was no significant change in ergosterol content in the control strain S1-G (∼6–8 mg/g DCW), and its final ergosterol yield was ∼100 mg/L (Figures 4B,C). In contrast, the engineered strain S1-G-AUAC produced little ergosterol in the first 32 h, whereas ergosterol was continuously accumulated until the steady-state (32–96 h). Finally, maximum ergosterol yiled in the shake-flask reached up to 421.1 mg/L with a content of 40.6 mg/g DCW after 72 h of cultivation (Figures 4B,C), which is 3.2 and 3.6 higher than that of S1-G, respectively. Overall, these results indicated that the expressions of the target genes were strictly controlled by the *GAL* regulatory network as anticipated. In addition, higher ergosterol production with comparable biomass was achieved in S1-G-AUAC owing to the decoupling of cell growth and ergosterol accumulation.

It is noteworthy that in addition to the promoted production of ergosterol in S1-G-AUAC, ergosta-5,7,22,24-tetraene-3β-ol, the direct precursor of ergosterol, was also accumulated to a rather high level (Figure 4D). The peak area of ergosta-5,7,22,24-tetraene-3β-ol in S1-G-AUAC was about 100-fold higher than that in the parent strain S1. With the adequate accumulation of intermediates of the ergosterol pathway, this recombinant industrial strain should be further engineered for optimal production of ergosterol as well as provided a potential alternative for the production of other closely related terpenoids such as brassinolide and 7-dehydrocholesterol.

### High-Level Production of Ergosterol via Fed-Batch Fermentation

**FIGURE 5:**
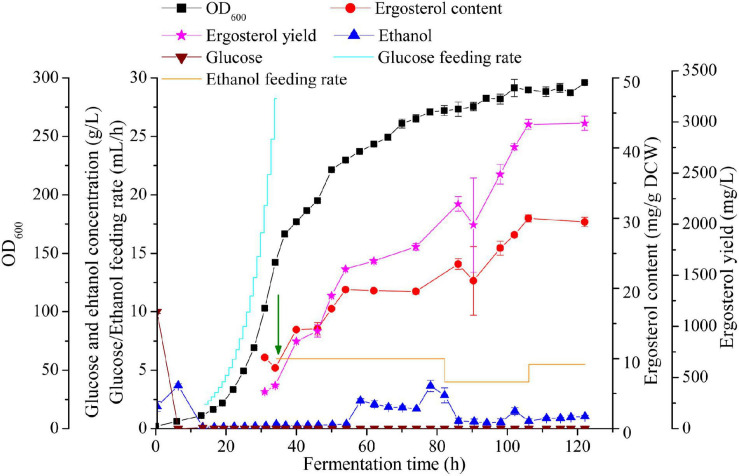
High cell density fermentation for ergosterol production with *S. cerevisiae* S1-G-AUAC in a 2-L bioreactor. Time courses of ergosterol production, cell growth, and ethanol and glucose concentration during the fed-batch fermentation. The arrow indicates that the feeding solution of glucose was replaced by ethanol.

The performance of the best ergosterol-producing strain (S1-G-AUAC) was evaluated with fed-batch fermentation in a 2 L bioreactor. Glucose and ethanol are commonly used carbon sources for production of terpenoids in yeast (Zhang et al., 2015; Qiao et al., 2019). Compared with glucose, ethanol can be utilized to directly supply cytosolic acetyl-CoA, resulting in significantly increased metabolic flux to the MVA pathway and accordingly terpenoid biosynthesis (De Jong-Gubbels et al., 1995; Ebert et al., 2018). In addition, NADPH generated from ethanol catabolism can provide sufficient reducing power for ergosterol biosynthesis in *S. cerevisiae*. However, when growing on ethanol, the rate of yeast biomass production is lower than that on glucose (Zampar et al., 2013). With the aim to develop an effective production process, a two-stage feeding process was employed: glucose pseudo-exponential feeding stage and ethanol feeding stage (Figure 5).

The profile of fed-batch fermentation indicated that the stages of cell growth and ergosterol accumulation were clearly separated by employing the two-stage feeding strategy (Figure 5). After 16 h cultivation, both the glucose in the batch medium and the ethanol produced was completely depleted. In pseudo-exponential feeding stage, glucose feeding rate was changed every 1 h according to the model until DO dropped to 15% and the agitation speed reached to its maximum (1,000 rpm). At the end of this feeding stage, OD_600_ reached 142.05 after 34 h fermentation, while ergosterol content remained at a low level (8.7 mg/g DCW). During the ethanol feeding stage, the production of ergosterol was greatly improved while the cell growth rate was decreased. The final cell density reached to 101.4 g/L (equivalent to OD_600_ of 296) after 122 h fermentation. At the end of fermentation, 2,986.7 mg/L ergosterol with a content of 29.5 mg/g DCW was obtained. Despite the significantly increased ergosterol yield, ergosterol content was lower than that using shaking flasks (40.6 mg/g DCW). This might be caused by the different growth conditions of cells in shake flasks and the fed-batch fermenter. During the whole fermentation process, about 101 g glucose and 372 g ethanol were fed, and the yield was 1.4 Cmol % (molar percentage of total substrate carbon added to the fermentation incorporated into ergosterol). As shown in Figure 5, high ethanol concentration was detected between 58 and 82 h, which was accompanied with the dramatically decreased ergosterol accumulation rate. The increment of ethanol concentration in the culture medium may lead to changes in the metabolic flux in the strain S1-G-AUAC, which is not beneficial for the synthesis of ergosterol. Therefore, the fermentation conditions should be further optimized to increase the production of ergosterol in yeast.

## Conclusion

In this work, combined metabolic engineering was established as an efficient approach to improve ergosterol productivity in an industrial *S. cerevisiae*. Ergosterol production was firstly increased via engineering of the pull and push of ergosterol biosynthesis by enhancing ergosterol esterification and systematically upregulating the transcription of ergosterol biosynthetic pathway genes. Then, ergosterol production was further improved by storage pool expansion through facilitating fatty acid biosynthesis. In addition, the decoupling of ergosterol production from cell growth further improved ergosterol production. A titer of 2986.7 mg/L ergosterol was eventually achieved using fed-batch fermentation by adopting a two-stage feeding strategy. The engineered yeast strains constructed in this work not only provides new ideas for enhancing ergosterol production but can also be used as a compelling platform for the production of other economically important terpenoids.

## Data Availability Statement

The original contributions presented in the study are included in the article/Supplementary Material, further inquiries can be directed to the corresponding author.

## Author Contributions

Z-JS and J-PL conceived the study. Z-JS performed the experiments, prepared figures and tables, and wrote the manuscript. J-ZL participated in data analysis, discussion, and revision of the manuscript. LZ and H-LX participated in the establishment of quantitative analysis method of ergosterol and squalene. Y-QJ participated in the fed-batch fermentation. G-SL participated in the revision of the manuscript. M-BW and L-RY participated in the discussion and coordination of the study. J-PL supervised the whole research and revised the manuscript. All authors read and approved the final manuscript.

## Conflict of Interest

The authors declare that the research was conducted in the absence of any commercial or financial relationships that could be construed as a potential conflict of interest.

## References

[B1] Aguilera F. Peinado R. Millan C. Ortega J. Mauricio J. (2006). Relationship between ethanol tolerance, H+-ATPase activity and the lipid composition of the plasma membrane in different wine yeast strains. *Int. J. Food Microbiol.* 110 34–42. 10.1016/j.ijfoodmicro.2006.02.002 16690148

[B2] Blaga A. C. Ciobanu C. Caşcaval D. Galaction A. (2018). Enhancement of ergosterol production by *Saccharomyces cerevisiae* in batch and fed-batch fermentation processes using n -dodecane as oxygen-vector. *Biochem. Eng. J.* 131 70–76. 10.1016/j.bej.2017.12.010

[B3] Da Silva N. A. Srikrishnan S. (2012). Introduction and expression of genes for metabolic engineering applications in *Saccharomyces cerevisiae*. *FEMS Yeast Res.* 12 197–214. 10.1111/j.1567-1364.2011.00769.x 22129153

[B4] Davies B. S. J. Wang H. S. Rine J. (2005). Dual activators of the sterol biosynthetic pathway of *Saccharomyces cerevisiae*: Similar activation/regulatory domains but different response mechanisms. *Mol. Cell. Biol.* 25 7375–7385. 10.1128/MCB.25.16.7375 16055745 PMC1190251

[B5] De Jong-Gubbels P. Vanrolleghem P. Heijnen S. Van Dijken J. P. Pronk J. T. (1995). Regulation of carbon metabolism in chemostat cultures of *Saccharomyces cerevisiae* grown on mixtures of glucose and ethanol. *Yeast* 11 407–418. 10.1002/yea.320110503 7597844

[B6] Donald K. A. Hampton R. Y. Fritz I. B. (1997). Effects of overproduction of the catalytic domain of 3-hydroxy-3-methylglutaryl coenzyme a reductase on squalene synthesis in *Saccharomyces cerevisiae*. *Appl. Environ. Microb.* 63 3341–3344. 10.1128/aem.63.9.3341-3344.1997 9292983 PMC168639

[B7] Ebert B. E. Czarnotta E. Blank L. M. (2018). Physiologic and metabolic characterization of *Saccharomyces cerevisiae* reveals limitations in the synthesis of the triterpene squalene. *FEMS Yeast Res.* 18: foy077. 10.1093/femsyr/foy077 30053028

[B8] Espenshade P. J. Hughes A. L. (2007). Regulation of sterol synthesis in eukaryotes. *Annu. Rev. Genet.* 41 401–427. 10.1146/annurev.genet.41.110306.130315 17666007

[B9] Gietz R. D. Schiestl R. H. (2007). High-efficiency yeast transformation using the LiAc/SS carrier DNA/PEG method. *Nat. Protoc.* 2 31–34. 10.1038/nprot.2007.13 17401334

[B10] Guo X. Xiao W. Wang Y. Yao M. Zeng B. Liu H. (2018). Metabolic engineering of *Saccharomyces cerevisiae* for 7-dehydrocholesterol overproduction. *Biotechnol. Biofuels* 11:192. 10.1186/s13068-018-1194-9 30026807 PMC6047132

[B11] He X. Huai W. Tie C. Liu Y. Zhang B. (2000). Breeding of high ergosterol-producing yeast strains. *J. Ind. Microbiol. Biotechnol.* 25 39–44. 10.1038/sj.jim.7000004

[B12] He X. P. Zhang B. R. Tan H. R. (2003). Overexpression of a sterol C-24(28) reductase increases ergosterol production in *Saccharomyces cerevisiae*. *Biotechnol. Lett.* 25 773–778. 10.1023/A:1023572403185 12882006

[B13] Heckman K. L. Pease L. R. (2007). Gene splicing and mutagenesis by PCR-driven overlap extension. *Nat. Protoc.* 2 924–932. 10.1038/nprot.2007.132 17446874

[B14] Heigwer F. Kerr G. Boutros M. (2014). E-CRISP: fast CRISPR target site identification. *Nat. Methods* 11 122–123. 10.1038/nmeth.2812 24481216

[B15] Henneberry A. L. Sturley S. L. (2005). Sterol homeostasis in the budding yeast, *Saccharomyces cerevisiae*. *Semin. Cell Dev. Biol.* 16 155–161. 10.1016/j.semcdb.2005.01.006 15797826

[B16] Jasinghe V. J. Perera C. O. (2005). Distribution of ergosterol in different tissues of mushrooms and its effect on the conversion of ergosterol to vitamin D2 by UV irradiation. *Food Chem.* 92 541–546. 10.1016/j.foodchem.2004.08.022

[B17] Jensen-Pergakes K. Guo Z. M. Giattina M. Sturley S. L. Bard M. (2001). Transcriptional regulation of the two sterol esterification genes in the yeast *Saccharomyces cerevisiae*. *J. Bacteriol.* 183 4950–4957. 10.1128/JB.183.17.4950-4957.2001 11489845 PMC95368

[B18] Karpova N. V. Andryushina V. A. Stytsenko T. S. Druzhinina A. V. Feofanova T. D. Kurakov A. V. (2016). A search for microscopic fungi with directed hydroxylase activity for the synthesis of steroid drugs. *Appl. Biochem. Microbiol.* 52 316–323. 10.1134/S000368381603008X 29509389

[B19] Kitchawalit S. Kanokmedhakul K. Kanokmedhakul S. Soytong K. (2014). A new benzyl ester and ergosterol derivatives from the fungus *Gymnoascus reessii*. *Nat. Prod. Res.* 28 1045–1051. 10.1080/14786419.2014.903478 24708569

[B20] Klug L. Daum G. (2014). Yeast lipid metabolism at a glance. *FEMS Yeast Res.* 14 369–388. 10.1111/1567-1364.12141 24520995

[B21] Li T. Liu G. Zhou W. Jiang M. Ren Y. Tao X. (2020). Metabolic engineering of *Saccharomyces cerevisiae* to overproduce squalene. *J. Agric. Food Chem.* 68 2132–2138. 10.1021/acs.jafc.9b07419 31989819

[B22] Lian J. Bao Z. Hu S. Zhao H. (2018). Engineered CRISPR/Cas9 system for multiplex genome engineering of polyploid industrial yeast strains. *Biotechnol. Bioeng.* 115 1630–1635. 10.1002/bit.26569 29460422

[B23] Livak K. J. Schmittgen T. D. (2001). Analysis of relative gene expression data using real-time quantitative PCR and the2-ΔΔC method. *Methods* 25 402–408. 10.1006/meth.2001.1262 11846609

[B24] Ma T. Shi B. Ye Z. Li X. Liu M. Chen Y. (2019). Lipid engineering combined with systematic metabolic engineering of *Saccharomyces cerevisiae* for high-yield production of lycopene. *Metab. Eng.* 52 134–142. 10.1016/j.ymben.2018.11.009 30471360

[B25] Meadows A. L. Hawkins K. M. Tsegaye Y. Antipov E. Kim Y. Raetz L. (2016). Rewriting yeast central carbon metabolism for industrial isoprenoid production. *Nature* 537 694–697. 10.1038/nature19769 27654918

[B26] Mendoza-Vega O. SabatiÃ J. Brown S. W. (1994). Industrial production of heterologous proteins by fed-batch cultures of the yeast *Saccharomyces cerevisiae*. *FEMS Microbiol. Rev.* 15 369–410. 10.1111/j.1574-6976.1994.tb00146.x 7848660

[B27] Paddon C. J. Westfall P. J. Pitera D. J. Benjamin K. Fisher K. McPhee D. (2013). High-level semi-synthetic production of the potent antimalarial artemisinin. *Nature* 496 528–532. 10.1038/nature12051 23575629

[B28] Parks L. W. Casey W. M. (1995). Physiological implications of sterol biosynthesis in yeast. *Annu. Rev. Microbiol.* 49 95–116. 10.1146/annurev.mi.49.100195.000523 8561481

[B29] Polakowski T. Bastl R. Stahl U. Lang C. (1999). Enhanced sterol-acyl transferase activity promotes sterol accumulation in *Saccharomyces cerevisiae*. *Appl. Microbiol. Biotechnol.* 53 30–35. 10.1007/s002530051610 10645622

[B30] Polakowski T. Stahl U. Lang C. (1997). Overexpression of a cytosolic hydroxymethylglutaryl-CoA reductase leads to squalene accumulation in yeast. *Appl. Microbiol. Biotechnol.* 49 66–71. 10.1007/s002530051138 9487712

[B31] Qi D. Scholthof K. G. (2008). A one-step PCR-based method for rapid and efficient site-directed fragment deletion, insertion, and substitution mutagenesis. *J. Virol. Methods* 149 85–90. 10.1016/j.jviromet.2008.01.002 18314204

[B32] Qiao J. Luo Z. Cui S. Zhao H. Tang Q. Mo C. (2019). Modification of isoprene synthesis to enable production of curcurbitadienol synthesis in *Saccharomyces cerevisiae*. *J. Ind. Microbiol. Biot.* 46 147–157. 10.1007/s10295-018-2116-3 30535727

[B33] Ro D. Paradise E. M. Ouellet M. Fisher K. J. Newman K. L. Ndungu J. M. (2006). Production of the antimalarial drug precursor artemisinic acid in engineered yeast. *Nature* 440 940–943. 10.1038/nature04640 16612385

[B34] Rodriguez S. Kirby J. Denby C. M. Keasling J. D. (2014). Production and quantification of sesquiterpenes in *Saccharomyces cerevisiae*, including extraction, detection and quantification of terpene products and key related metabolites. *Nat. Protoc.* 9 1980–1996. 10.1038/nprot.2014.132 25058645

[B35] Rodwell V. W. Nordstrom J. L. Mitschelen J. J. (1976). Regulation of HMG-CoA reductase. *Adv. Lipid Res.* 14 1–74. 10.1016/B978-0-12-024914-5.50008-5 769497

[B36] Scalcinati G. Partow S. Siewers V. Schalk M. Daviet L. Nielsen J. (2012). Combined metabolic engineering of precursor and co-factor supply to increase α-santalene production by *Saccharomyces cerevisiae*. *Microb. Cell Fact.* 11:117. 10.1186/1475-2859-11-117 22938570 PMC3527295

[B37] Scodelaro Bilbao P. G. Garelli A. Díaz M. Salvador G. A. Leonardi P. I. (2020). Crosstalk between sterol and neutral lipid metabolism in the alga *Haematococcus pluvialis* exposed to light stress. *Biochim. Biophys. Acta (BBA) Mol. Cell Biol. Lipids* 1865:158767. 10.1016/j.bbalip.2020.158767 32736090

[B38] Shin G. Veen M. Stahl U. Lang C. (2012). Overexpression of genes of the fatty acid biosynthetic pathway leads to accumulation of sterols in *Saccharomyces cerevisiae*. *Yeast* 29 371–383. 10.1002/yea.2916 22926964

[B39] Subbiah M. T. R. Abplanalp W. (2003). Ergosterol (major sterol of baker’s and brewer’s yeast extracts) inhibits the growth of human breast cancer cells in vitro and the potential role of its oxidation products. *Int. J. Vitam. Nutr. Res.* 73 19–23. 10.1024/0300-9831.73.1.19 12690907

[B40] Sun Y. Sun L. Shang F. Yan G. (2016). Enhanced production of β-carotene in recombinant *Saccharomyces cerevisiae* by inverse metabolic engineering with supplementation of unsaturated fatty acids. *Process Biochem.* 51 568–577. 10.1016/j.procbio.2016.02.004

[B41] Tokuhiro K. Muramatsu M. Ohto C. Kawaguchi T. Obata S. Muramoto N. (2009). Overproduction of geranylgeraniol by metabolically engineered *Saccharomyces cerevisiae*. *Appl. Environ. Microb.* 75 5536–5543. 10.1128/AEM.00277-09 19592534 PMC2737941

[B42] Veen M. Lang C. (2005). Interactions of the ergosterol biosynthetic pathway with other lipid pathways. *Biochem. Soc. Trans.* 33 1178–1181. 10.1042/BST20051178 16246076

[B43] Wang S. Q. Wang T. Liu J. F. Deng L. Wang F. (2018). Overexpression of Ecm22 improves ergosterol biosynthesis in *Saccharomyces cerevisiae*. *Lett. Appl. Microbiol.* 67 484–490. 10.1111/lam.13061 30098030

[B44] Westfall P. J. Pitera D. J. Lenihan J. R. (2012). Production of amorphadiene in yeast, and its conversion to dihydroartemisinic acid, precursor to the antimalarial agent artemisinin. *Proc. Natl Acad. Sci. U.S.A.* 109 111–118. 10.1073/pnas.1110740109 22247290 PMC3271868

[B45] Wu G. Yan Q. Jones J. A. Tang Y. J. Fong S. S. Koffas M. A. G. (2016). Metabolic burden: cornerstones in synthetic biology and metabolic engineering applications. *Trends Biotechnol.* 34 652–664. 10.1016/j.tibtech.2016.02.010 26996613

[B46] Wu H. F. Li Y. L. Song G. M. Xue D. H. (2012). Producing ergosterol from corn straw hydrolysates using *Saccharomyces cerevisiae*. *Afr. J. Biotechnol.* 11 11160–11167. 10.5897/AJB11.1116

[B47] Xie W. Liu M. Lv X. Lu W. Gu J. Yu H. (2014). Construction of a controllable β-carotene biosynthetic pathway by decentralized assembly strategy in *Saccharomyces cerevisiae*. *Biotechnol. Bioeng.* 111 125–133. 10.1002/bit.25002 23860829

[B48] Yang H. Bard M. Bruner D. A. Gleeson A. Deckelbaum R. J. Aljinovic G. (1996). Sterol esterification in yeast: a two-gene process. *Science* 272 1353–1356. 10.1126/science.272.5266.1353 8650549

[B49] Yang H. Tong J. Lee C. W. Ha S. Eom S. H. Im Y. J. (2015). Structural mechanism of ergosterol regulation by fungal sterol transcription factor Upc2. *Nat. Commun.* 6 6129–6141. 10.1038/ncomms7129 25655993

[B50] Zampar G. G. Kümmel A. Ewald J. Jol S. Niebel B. Picotti P. (2013). Temporal system-level organization of the switch from glycolytic to gluconeogenic operation in yeast. *Mol. Syst. Biol.* 9:651. 10.1038/msb.2013.11 23549479 PMC3693829

[B51] Zavrel M. Hoot S. J. White T. C. (2013). Comparison of sterol import under aerobic and anaerobic conditions in three fungal species, *Candida albicans*, *Candida glabrata*, and *Saccharomyces cerevisiae*. *Eukaryotic Cell* 12 725–738. 10.1128/EC.00345-12 23475705 PMC3647772

[B52] Zhang G. Cao Q. Liu J. Liu B. Li J. Li C. (2015). Refactoring β-amyrin synthesis in *Saccharomyces cerevisiae*. *Aiche J.* 61 3172–3179. 10.1002/aic.14950

[B53] Zhang Y. Nielsen J. Liu Z. (2017). Engineering yeast metabolism for production of terpenoids for use as perfume ingredients, pharmaceuticals and biofuels. *FEMS Yeast Res.* 17:x80. 10.1093/femsyr/fox080 29096021

[B54] Zhao W. Hang B. Zhu X. Wang R. Shen M. Huang L. (2016). Improving the productivity of S-adenosyl-L-methionine by metabolic engineering in an industrial *Saccharomyces cerevisiae* strain. *J. Biotechnol.* 236 64–70. 10.1016/j.jbiotec.2016.08.003 27510807

[B55] Zweytick D. Leitner E. Kohlwein S. D. Yu C. Rothblatt J. Daum G. (2000). Contribution of Are1p and Are2p to steryl ester synthesis in the yeast *Saccharomyces cerevisiae*. *Eur. J. Biochem.* 267:1075–1082. 10.1046/j.1432-1327.2000.01103.x 10672016

