# Supplementary material for: Combined Biosynthetic Pathway Engineering and Storage Pool Expansion for High-Level Production of Ergosterol in Industrial Saccharomyces cerevisiae
Source: Front Bioeng Biotechnol. 2021 Jun 29;9:681666. doi: 10.3389/fbioe.2021.681666 (PMC8275991; doi:10.3389/fbioe.2021.681666)
Supplement: Supplementary file 1 [file Data_Sheet_1.docx]

Supplementary Material

Combined Biosynthetic Pathway Engineering and Storage Pool Expansion for High-level Production of Ergosterol in Industrial *Saccharomyces cerevisiae*

Zhi-Jiao Sun, Jia-Zhang Lian, Li Zhu, Yi-Qi Jiang, Guo-Si Li, Hai-Long Xue, Mian-Bin Wu, Li-Rong Yang, Jian-Ping Lin^*^

Key Laboratory of Biomass Chemical Engineering of Ministry of Education, College of Chemical and Biological Engineering, Zhejiang University, Hangzhou, China

*** Correspondence:**Corresponding Author
Email: linjp@zju.edu.cn

**Fig. S1.** Squalene production in yeast strains S1, S1-tHMG1, and S1-tHA in 250 mL shake flasks with 50 mL of YPD medium containing 50 g/L glucose. The squalene production was quantified after 48 h cultivation.

**Fig. S2.** The effect of overexpression of *tHMG1* on the production of ergosterol. **(A)** Cell growth curves in strains with *tHMG1* overexpressed; **(B)** Comparison of ergosterol production in strains S1/S1-tHMG1, S1-ARE2/S1-tHA, and S1-AU/S1-tHAU.

**Fig. S3.** Analysis of the relative transcriptional levels of the targets genes under the control of constitutive promoters in recombinant strains. The transcriptional levels of *tHMG1* gene were compared between the starting strain S1 and the recombinant strain S1-tHMG1, while the transcriptional levels of *ARE2*, *UPC2-1,* and *ACC1* genes were compared between the starting strain S1 and recombinant strain S1-AUAC. Cells were grown in 250 mL shake flasks with 50 mL of YPD medium containing 50 g/L glucose and were collected after 48 h cultivation.

**Fig. S4.** Dissolved oxygen profiles in the two-stage feeding process.

**Table S1.** List of primers used in the study.

| Primers | Sequence (5’-3’) |
| --- | --- |
| HO-N20F | GCCGGCTTGATCGACTCAGAgttttagagctagaaatagcaagttaaaataaggc |
| HO-N20R | TCTGAGTCGATCAAGCCGGCgatcatttatctttcactgcggagaa |
| Ty4-N20F | GTATGTAGGCGTTCTGTAAGgttttagagctagaaatagcaagttaaaataaggctagtccg |
| Ty4-N20R | CTTACAGAACGCCTACATACgatcatttatctttcactgcggagaagtttc |
| Ty3-N20F | GGTAGTTCAGCTAAGGGACGgttttagagctagaaatagcaagttaaaataaggc |
| Ty3-N20R | CGTCCCTTAGCTGAACTACCgatcatttatctttcactgcggagaag |
| PAc-N20F | GCAAATGGCGATTGCAAATTgttttagagctagaaatagcaagttaaaataaggc |
| PAc-N20R | AATTTGCAATCGCCATTTGCgatcatttatctttcactgcggagaag |
| GAL80 N20F | GTCGGTCTCAACGCAGCCAAgttttagagctagaaatagcaagttaaaataaggc |
| GAL80 N20R | TTGGCTGCGTTGAGACCGACgatcatttatctttcactgcggagaag |
| HO.U-PPGK1F | TCCCTGAATTTATGTACGGCGAGCATATAGAAGTTCGTGAAGCATTCTTAattttagattcctgacttcaac |
| PPGK1-tHR | ATTGGTCCATAGTtgttttatatttgttgtaaaaagtagataattacttccttgatgatc |
| tHMG1F | TTTACAACAAATATAAAACAactatggaccaattggtgaaaactgaagtcacc |
| tHMG1R | ATAAATCATAAGAAATTCGCacgtaacacatggtgctgttgtg |
| TADH1-tHF | AACAGCACCATGTGTTACGTgcgaatttcttatgatttatgatttttattattaaataag |
| HO.D-TADH1R | AGTTTGAATTGCTATTTTATAAGATTCAGGGCCTTCGCCCTTTTTCACAAtagaggtgtggtcaataagag |
| dor.HO-tHF | tccctgaatttatgtacggcgagc |
| dor.HO-tHR | agtttgaattgctattttataagattcagggcct |
| Ty4.U-PPGK1F | AGCGTAACAATGCATACTGACATTATAAGCCTGAACATTACGAGTTTAAGattttagattcctgacttcaac |
| HO-tH-confF | tgaaaacaggaagacaaggaatttgagg |
| HO-tH-confR | cctggtcgtcacagtagctgacata |
| PPGK1-ARR | GATCCTTCTTCTTGTCCATtgttttatatttgttgtaaaaagtagataattac |
| ARE2F | TTTACAACAAATATAAAACAatggacaagaagaaggatctactggagaa |
| ARE2R | ATAAATCATAAGAAATTCGCttagaatgtcaagtacaacgtacacat |
| TADH1-ARF | GTTGTACTTGACATTCTAAgcgaatttcttatgatttatgatttttattat |
| Ty4.D-TADH1R | AGAAGTAGCTTTCGCAAATACACCAATGCATTCGTTGATAATTTACGCAGtagaggtgtggtcaataaga |
| dor.Ty4-ARE2F | agcgtaacaatgcatactgacattataagcc |
| dor.Ty4-ARE2R | agaagtagctttcgcaaatacaccaatg |
| Ty4-AR-confF | agatccatattcaaccttcattaatactgcc |
| Ty4-AR-confR | agttatcaccaaatcagttgagcagatcac |
| pRS42HF | gcggatctcttatgtctttacgatttatagttttcatt |
| pRS42HR | catctagaaaacttagattagattgctatgctttctttctaat |
| UPC2F | TAATCTAAGTTTTCTAGATGatgagcgaagtcggtatacagaatcac |
| UPC2R | TAAAGACATAAGAGATCCGCtcataacgaaaaatcagagaaatttgttgttgtcat |
| UPC2-1F | acagtggaggtggtgAtatgcatatgatgct |
| UPC2-1R | Tcaccacctccactgtattcgtcaac |
| pU-PTEF1-UF | ttttctagatgagcgaagtcggtatacaga |
| pU-PTEF1-DR | cacgtagtgggccatcgc |
| PACT1-PTEF1F | CGATGGCCCACTACGTGcaatatttctctgtcacccggc |
| PACT1-PTEF1R | GACTTCGCTCATCTAGAAAAtgttaattcagtaaattttcgatcttgggaag |
| Ty3.U-PACT1F | CGGAATCTCGGACCTAAACTAATTGTTCAGGCATTCGTACGTTTGGGTAGcaatatttctctgtcacccggcc |
| Ty3.D-TADH2R | TGAAACAACTTTATAACAAAGCGAACAAAATGGGCAACATGAGATGAAACcggccgctctagaactagtatga |
| dor.Ty3-UPC2-1F | cggaatctcggacctaaactaat |
| dor.Ty3-UPC2-1R | tgaagcaaccttataacaaagcgaac |
| Ty3-UPC2-1-confF | ctgcaagagtgtatctcgggaac |
| Ty3-UPC2-1-confR | gtcagctacacgcaaattctgtg |
| PACC1 HUF | cgttgtgccaacaagtcgca |
| PACC1 HUR | GAAGCTATGattgaagagagcagaacaattgtagaacc |
| PTEF1-PACC1F | CTGCTCTCTTCAATcatagcttcaaaatgtttctactcc |
| PTEF1-PACC1R | CTTCGCTCATCtagaaaacttagattagattgctatgc |
| PACC1 HDF | TCTAAGTTTTCTAGatgagcgaagaaagcttattcgag |
| PACC1 HDR | ttatttgctatcaggatcttggatatg |
| PT1-PA1-confF | gaacaacgcccaacaatttccac |
| PT1-PA1-confR | atcaatgggaggtcatcgaaagag |
| GAL80 HUF | acctcctccagatggaatcccttc |
| GAL80 HUR | GCACTGGGGGattttgaggtcgttcgggcgag |
| GAL80 HDF | ACCTCAAAATcccccagtgcagcgaacgttata |
| GAL80 HDR | ccatgctaccttccatggttgagc |
| Δgal80 confF | ggtcttctggtctgttagggc |
| Δgal80 confR | gggagcgcaatttactaatggc |
| PGAL1F | tgaagtacggattagaagccgc |
| PGAL1R | tatagttttttctccttgacgttaaagtat |
| PG1-ARE2F | GTCAAGGAGAAAAAACTATAatggacaagaagaaggatctactgg |
| PG1-ARE2R | GGCTTCTAATCCGTACTTCActtaaactcgtaatgttcaggct |
| PG1-UPC2-1F | GTCAAGGAGAAAAAACTATAatgagcgaagtcggtatacagaat |
| PG1-UPC2-1R | GGCTTCTAATCCGTACTTCActacccaaacgtacgaatgcct |
| HO.U-PGAL1F | TCCCTGAATTTATGTACGGCGAGCATATAGAAGTTCGTGAAGCATTCTTAtgaagtacggattagaagccgc |
| HO.D-TADH2R | AGTTTGAATTGCTATTTTATAAGATTCAGGGCCTTCGCCCTTTTTCACAAcggccgctctagaactagtatg |
| PG1-PT1F | GTCAAGGAGAAAAAACTATAatgagcgaagaaagcttattcg |
| PG1-PT1R | GGCTTCTAATCCGTACTTCAattgaagagagcagaacaattgt |
| qACT1F | atgcaaaccgctgctcaatc |
| qACT1R | caataccggcagattccaaacc |
| qtHMG1F | aaaccagctgccatcaactg |
| qtHMG1R | gctgcatgtgcgttaaatcc |
| qARE2 F | attggctgtgcgcaattctg |
| qARE2 R | gccacacagttcaaaatggc |
| qUPC2-1F | tgcgttccaacttcagcaac |
| qUPC2-1R | attcccgtggattgcaatgc |
| qACC1F | accattgtcgctcaaagtgc |
| qACC1R | accatcttcaggagaggtacaac |

The sequences of the primers used for ampliﬁcation of the DNA fragments are shown in lower cases, while the overhangs in capital letters are used for the introduction of specific 20-bp target sequences for gRNAs or served as homologous arms for overlap extension PCR or [homologous recombination](https://www.sciencedirect.com/topics/agricultural-and-biological-sciences/homologous-recombination) based genome integration. The capital letters in the primers of UPC2-1F and UPC2-1R were used for the introduction of point mutations.

**Table S2.** List of plasmids used in the study.

| Plasmids | Description | Reference |
| --- | --- | --- |
| p42H-SpCas9 | pRS425-*P_TEF1_*-SpCas9-*T_ADH2_*, *LEU2* replaced with *HygB* | (Lian, 2018) |
| pKan100-ADE2.1 | pRS423*-*P_SNR52_*-ADE2.1-SpSgRNA-*T_SUP4_*, *HIS3* replaced with *P_TEF1_*(100bp)-KanMX | (Lian, 2018) |
| pTar-HO | pKan100-ADE2.1, ADE2.1 was replaced by N20 sequence targeting *HO* site | this study |
| pTar-Ty4 | pKan100-ADE2.1, ADE2.1 was replaced by N20 sequence targeting *Ty4* site | this study |
| pTar-Ty3 | pKan100-ADE2.1, ADE2.1 was replaced by N20 sequence targeting *Ty3* site | this study |
| pTar-PAc | pKan100-ADE2.1, ADE2.1 was replaced by N20 sequence targeting *ACC1* promoter | this study |
| pTar-G80 | pKan100-ADE2.1, ADE2.1 was replaced by N20 sequence targeting *GAL80* site | this study |
| pEASY | *pEASY*®-Blunt Simple Cloning Vector | TransGen Biotech |
| pEASY-dor-*tHMG1* | pEASY-*HO.U-P_PGK1_-tHMG1-T_ADH1_-HO.D* | this study |
| pEASY-dor-*ARE2* | pEASY-*Ty4.U-P_PGK1_-ARE2-T_ADH1_-Ty4.D* | this study |
| p42H-*P_TEF1_-UPC2* | p42H-*P_TEF1_-UPC2-T_ADH2_* | this study |
| p42H-*P_TEF1_-UPC2-1* | p42H-*P_TEF1_-UPC2-1-T_ADH2_* | this study |
| p42H-*P_ACT1_-UPC2-1* | p42H-*P_ACT1_-UPC2-1-T_ADH2_* | this study |
| pEASY-dor-*UPC2-1* | pEASY-*Ty3.U-P_ACT1_-UPC2-1-T_ADH2_-Ty3.D* | this study |
| pEASY-dor-*Δgal80* | pEASY-*GAL80.U-GAL80.D* | this study |
| pEASY-dor-*PTEF1* | pEASY-*P_ACC1_.U-PTEF1-P_ACC1_.D* | this study |
| pEASY-dor-G*ARE2* | pEASY-*Ty4.U-P_GAL1_-ARE2-T_ADH1_-Ty4.D* | this study |
| pEASY-dor-G*UPC2-1* | pEASY-*HO.U-P_GAL1_-UPC2-1-T_ADH2_-HO.D* | this study |
| pEASY-dor-*P_GAL1_* | pEASY-*P_ACC1_.U-P_GAL1_-P_ACC1_.D* | this study |

**Reference**

Lian, J., Bao, Z., Hu, S., and Zhao, H. (2018). Engineered CRISPR/Cas9 system for multiplex genome engineering of polyploid industrial yeast strains. Biotechnol. Bioeng. 115, 1630-1635. doi: 10.1002/bit.26569.
